# Taillessness in a Cloned Cow is Not Genetically Transmitted

**DOI:** 10.1089/cell.2017.0018

**Published:** 2017-12-01

**Authors:** Stefan Wagner, Alison Cullum, David N. Wells, Götz Laible

**Affiliations:** ^1^ AgResearch Limited, Ruakura Research Centre, Hamilton, New Zealand.; ^2^ Current address: Rowett Institute, Aberdeen, United Kingdom.

**Keywords:** taillessness, cloning, transgenic, cattle, epigenetic, anury

## Abstract

Somatic cell nuclear transfer (SCNT), commonly referred to as cloning, results in the generation of offspring that, except for mitochondrial DNA, are genetically identical to the nuclear donor. We previously used a genetically modified bovine cell line as the donor for SCNT and obtained a calf, named Daisy, that was born without a tail. To determine whether the missing tail was a result of the genetic modification, we performed recloning experiments by using either cells from a sacrificed pregnancy of a second clone (Daisy's “twin” clone) or cells from tailless Daisy as donors for SCNT. Cloned fetuses from aborted pregnancies and a cloned live calf that died shortly after birth were examined and confirmed to all possess tails. Hence, the observed phenotype of Daisy's lacking tail is not due to the introduced transgene or a mutation present in the cell that was used for her production. Rather, the missing tail has most likely arisen from an epigenetic reprogramming error during development.

## Introduction

Taillessness (anury) in cattle is a rare congenital defect. Statistical analysis of a study with 25 tailless animals of different breeds suggested an occurrence of between one in 3000 and one in 20,500 (Huston and Wearden, 1958). Similarly, investigations of tailless cattle in Germany indicated frequencies of one in 23,800 (Rieck, 1966) and one in 17,700 (Kloppert, 1986). Results of a brief study of 50 tailless cows by Gilmore and Fechheimer (1957) suggest that taillessness in cattle is neither a simple dominant nor a simple recessive trait, an assumption that was also inferred from test matings of tailless animals (Greene et al., 1973).

This apparent lack of heritability contrasts with taillessness in Manx cats that is inherited through a dominant and homozygous lethal allele of the Manx *M* gene (Robinson, 1993) or in a reported mutated house mouse strain where a tailless phenotype was transmitted through two dominant and homozygous lethal alleles (Chesley and Dunn, 1936). In a case study, Lotfi and Shahryar (2009) described the occurrence of a tailless calf in Iran but the underlying cause for the taillessness was not determined. A case of taillessness was also reported in the highly inbred herd of Chillingham Wild White cattle that have been genetically isolated for several centuries (Ingham and Widdows, 2005).

In this study, we show that recloning of a tailless transgenic calf yields fetuses and a live calf with tails, which suggests that the originally observed taillessness is not of genetic origin.

## Materials and Methods

### Somatic cell nuclear transfer

Somatic cell nuclear transfer (SCNT) was performed by using a zona-free method as previously described (Jabed et al., 2012; Oback et al., 2003).

### Derivation of Twin 312/3 and Daisy 312/3 cells

After SCNT using cell line 312/3 as donor, a day 65 fetus was recovered in its amniotic sac from the slaughter-sacrificed recipient (Jabed et al., 2012). The fetus was briefly rinsed in 70% ethanol and washed twice in phosphate buffered saline (PBS). After removal of head, tail, and visceral organs, the fetus was cut into small pieces that were digested with 0.05% trypsin for about 1 hour while adding fresh trypsin every 10 minutes. Digested cells were spun down, and the resuspended cells were cultured in Dulbecco's modified Eagle's medium (DMEM)/F12 + 10% fetal calf serum + antibiotic/anti-mycotic. Expanded cells, called Twin 312/3, were cryopreserved for future use.

Daisy 312/3 cells were derived from an ear biopsy of Daisy. The ear biopsy was shaved to remove hair, briefly rinsed in 70% ethanol, and washed twice with PBS. The tissue was then cut to expose and remove the inner cartilage layer. The remaining tissue was finely diced into small tissue blocks with scalpel blades and then explanted in a tissue culture dish. Individual tissue pieces were covered with culture medium (DMEM/F12, 10% fetal calf serum) complemented with one times Antibiotic-Antimycotic (Gibco). After 3 days, the medium was replaced with culture medium containing one times Penicillin-Streptomycin (Gibco). Cellular outgrowth was observed within 5 days, and cells were further cultured until day 12 and then cryopreserved.

### Polymerase chain reaction genotyping

Presence of the transgene was determined by polymerase chain reaction amplification as previously described (Jabed et al., 2012).

### Skeletal bone preparation

The posterior end of the vertebral column including the hip was cut out from the carcass after euthanasia of Daisy by an overdose of sodium pentobarbitone at close to 4 years of age. After dissection and removal of the tissue associated with the bone structure, the skeletal section was buried under a concrete basin. After 6 months, the skeletal section was recovered and cleaned to remove any remaining tissue. For comparison, a similar skeletal section was prepared from a wild-type Hereford × Friesian cow. The bone structures of the two cows were then visually compared to determine anatomical differences between Daisy and the wild-type cow.

### Animal studies

All animal studies were undertaken in compliance with New Zealand laws covering the use of animals for research and were approved by the Environmental Protection Agency, New Zealand, and the Ruakura Animal Ethics Committee.

## Results

**FIG. 1.**
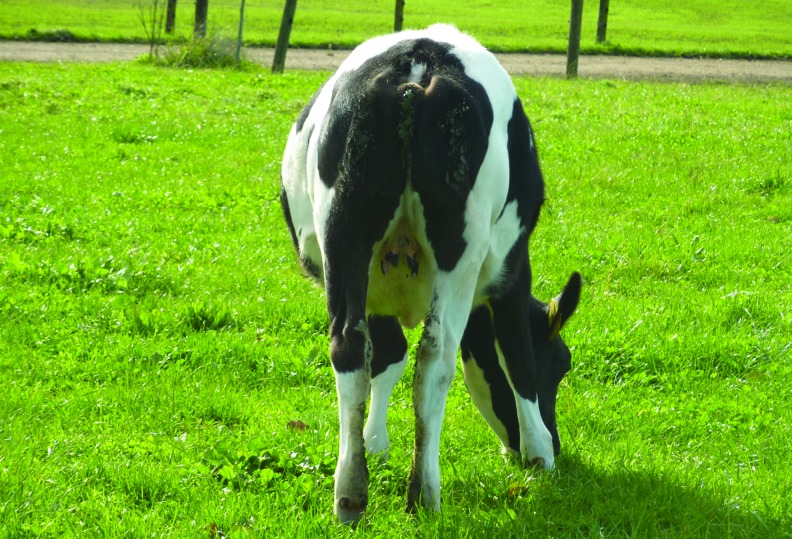
Daisy at the age of 7 months. View of the posterior end showing the absence of a tail. Color images available online at www.liebertpub.com/cell

We previously reported the production of a transgenic calf, Daisy, that was generated by SCNT using a donor cell from the clonal cell line 312/3 engineered with a tandem micro RNA cassette for the knockdown of the expression of an allergy causing milk protein (Jabed et al., 2012). When the calf was born, it had a very noticeable congenital defect, which was the lack of a tail (Fig. 1). The underlying cause for the taillessness was unknown and led us to investigate the tailless phenotype of this transgenic cattle line.

### Characterization of anatomical abnormalities

**FIG. 2.**
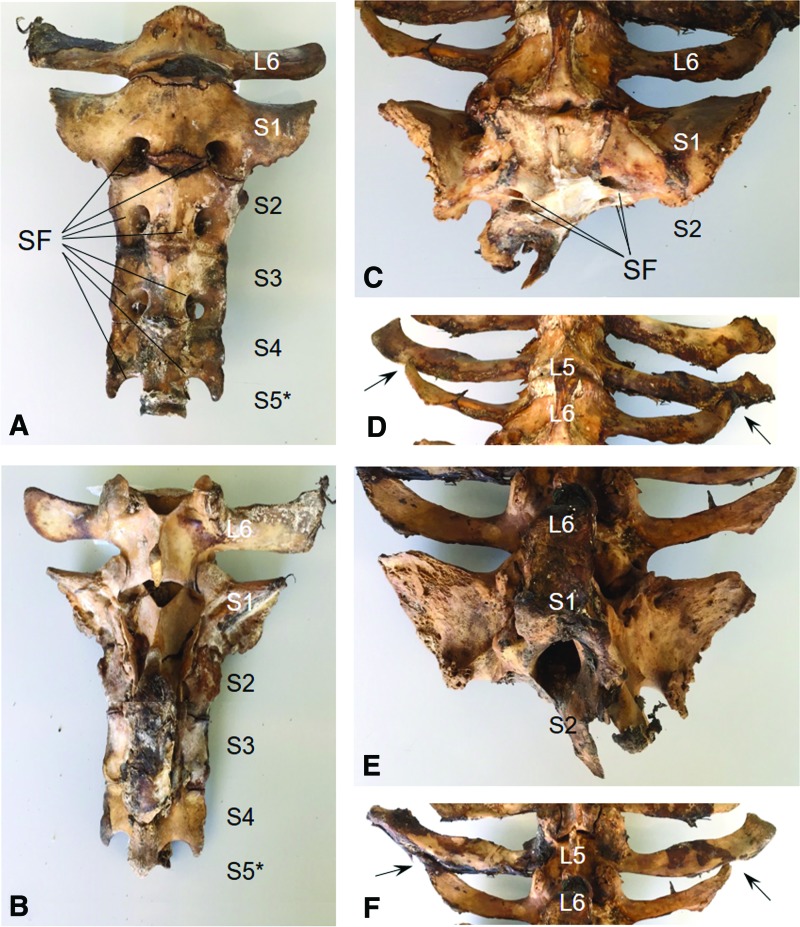
Posterior skeletal section. Shown is the ventral view of the sacrum with parts of the adjacent lumbar section of a wild-type cow **(A)** and the tailless cow Daisy **(C)**. The sixth lumbar (L6) and five sacral (S1–S5) vertebrae and the position of the observed sacral foraminae (SF) are indicated. Dorsal views of the sacrum are depicted in panels (**B**, wild type) and (**E**, Daisy). Panels **(D)** and **(F)** show ventral and dorsal views of Daisy's overlapping transverse processes of the L6 and L5 vertebrae (arrows). S5* denotes the normal position of the fifth sacral vertebra, S5, which is not shown for the wild-type sacrum. Color images available online at www.liebertpub.com/cell

Daisy had a normal number of lumbar vertebrae (six) with deviations from a normal anatomical structure evident in the posterior vertebra. The sacrum appeared to consist of two fused vertebrae instead of the fusion of five sacral vertebrae that usually form the sacrum (Fig. 2). This was supported by only two ventral openings (sacral foraminae) on each side (Fig. 2C) compared with the usual four openings on each side in a normal bovine sacrum (Fig. 2A). Moreover, the sacrum of the tailless cow had a pronounced asymmetry that was apparent in the transverse processes of the sixth lumbar vertebrae. These processes were unevenly aligned with the long axis of the vertebral body and orientated cranially, overlapping the processes of the preceding vertebra delineating an abnormal anatomy (Fig. 2D, F).

### Recloning with rejuvenated 312/3 cells

**FIG. 3.**
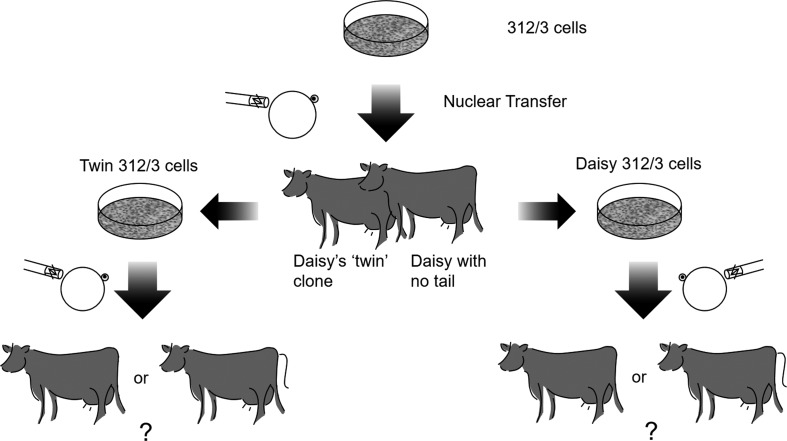
Schematic overview of the experimental somatic cell nuclear transfer strategy to determine the underlying cause for the missing tail as genetic or epigenetic. Shown is the genealogy of clonal cell lines 312/3, Twin 312/3 and Daisy 312/3 and the cloning and recloning steps undertaken to produce Daisy, Daisy's twin, and their cloned offspring.

To elucidate the underlying cause of the missing tail, we decided to reclone Daisy. For the recloning, we followed a two-pronged approach. Since the original 312/3 cells were no longer available and to get access to rejuvenated 312/3 cells with rejuvenated growth potential for future cloning and biobanking, we sacrificed a healthy “twin” fetus of Daisy at day 65 of gestation to rederive 312/3 cells (Materials and Methods, Fig. 3). At this time, Daisy was not yet born and, hence, we were not aware of her taillessness. Although we did not notice anything abnormal with Daisy's twin fetus that was used for the rederivation of 312/3 cells, we have no photographic evidence as to whether or not the twin fetus had a tail (Fig. 3).

To determine whether the single cell that was used to produce Daisy's twin fetus had the developmental potential for a tail, we used rederived Twin 312/3 cells for SCNT. The resulting cloned fetuses were then examined for the presence or absence of a tail in the recloned twins.

**FIG. 4.**
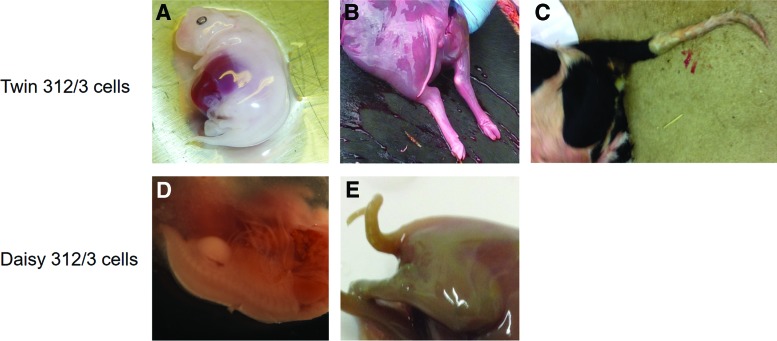
Cloned progeny with tails generated from two sources of rederived 312/3 donor cells. Shown are pictures documenting the tails of two cloned fetuses recovered at day 43 **(A)** and day 171 **(B)** of gestation and a cloned calf born at day 270 of gestation **(C)**, all generated with Twin 312/3 donor cells as indicated. Panels **(D)** and **(E)** depict cloned fetuses generated with Daisy 312/3 cells that were recovered at day 42 **(D)** and day 75 **(E)** of gestation. Color images available online at www.liebertpub.com/cell

First, we aborted a healthy pregnancy at day 43 and recovered a fetus that displayed a normal-looking tail (Fig. 4A). Two more pregnancies were allowed to develop to term. One of the recipients developed hydrops, and the pregnancy was aborted at day 171 of gestation. The recovered fetus displayed a tail of normal appearance (Fig. 4B). The final pregnancy went to term, and a calf was born on day 270 of gestation after induced parturition. The calf had a normal tail (Fig. 4C) but died 30 minutes after delivery due to non-closure of the valve separating right and left side of the heart postnatally, which resulted in circulatory and oxygenation failure.

Twin 312/3 cells and cells from the tailless calf Daisy originate from the same 312/3 cell clone and, thus, ought to have identical genomes. To discount the potential occurrence of a spontaneous somatic mutation in the single cell used to produce Daisy that might have caused the taillessness phenotype, we next rederived cells from an ear biopsy of Daisy (Fig. 3). These Daisy 312/3 cells are direct progenitors of Daisy's single SCNT donor cell and, thus, would harbor the same somatic mutation. Daisy 312/3 cells were then used for SCNT to produce clones that could be examined for the presence or absence of a tail (Fig. 3).

**FIG. 5.**
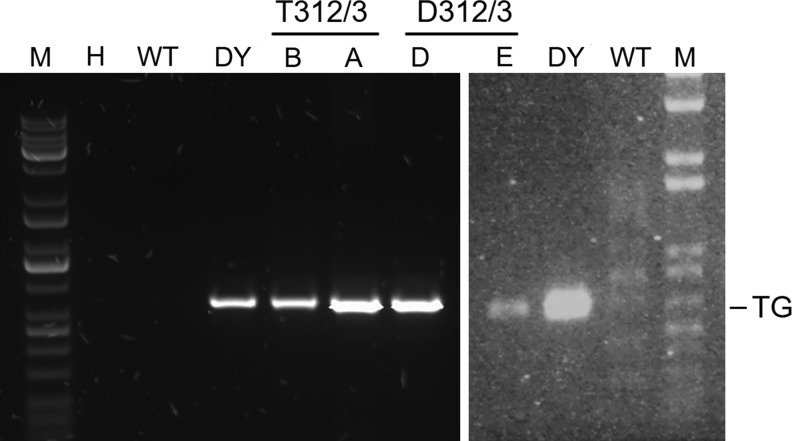
PCR detection of the transgene in recloned fetuses. PCR result for the amplification of a transgene-specific fragment with genomic DNA isolated from wild-type bovine fetal fibroblasts (WT), Daisy (DY), fetuses shown in Figure 4A, B generated with Twin 312/3 donor cells (T312/3, A and B) and fetuses shown in Figure 4D, E generated with Daisy 312/3 cells (D321/3, D and E). H, water control; M, size marker; PCR, polymerase chain reaction.

We obtained two fetuses from aborted pregnancies and observed that both a day 43 and a day 75 fetus possessed tails (Fig. 4D, E). The day 75 fetus had a kinked tail that had been observed occasionally earlier in cloned bovine fetuses (A. Ledgard, AgResearch, Ruakura Research Centre, pers. comm.). All fetuses were genotyped and confirmed to possess the transgene (Fig. 5).

## Discussion

Sexual reproduction typically produces genetically different offspring, and these genetic differences are the underlying cause for phenotypic differences between individuals of the same species. Clones, in contrast, share the same nuclear genetics, and any phenotypic differences between them are due to either the accrual of somatic mutations during development or environmental causes that are broadly referred to as epigenetics. Teasing apart genetic from epigenetic causes is not straightforward, and some phenotypic differences that have been ascribed to epigenetic causes may instead more likely be due to as-yet-uncharacterized genetic differences.

In this context, the occurrence of a clone calf without a tail presented us with the serendipitous opportunity to unequivocally determine whether the observed rare phenotype is of genetic or epigenetic origin. For the generation of Daisy by SCNT, we used a genetically modified donor cell and we, thus, had to determine whether or not Daisy's taillessness was a result of the chromosomal transgene insertion. Recloning, using cells derived from Daisy's twin fetus (Twin 312/3), allowed us to generate two fetuses and a live-born calf with the same genetic background and, thus, enabled us to discern genetic and epigenetic causes of the tail phenotype.

Both the fetuses and the calf had clearly discernable tails. Presuming that the initially used clonal cell line 312/3 and its Twin 312/3 derivative are genetically identical, this demonstrates that the genome of these cells retains the full competence for the development of a tail and indicates that Daisy's missing tail was not a consequence of the integration of the transgene into a random chromosomal locus. Moreover, the two fetuses generated with Daisy 312/3 cells derived from an ear biopsy of Daisy also had tails. This essentially excludes the presence of a spontaneous mutation in the genome of Daisy's cells that originated from the single cell used to generate her as an underlying cause for the missing tail.

Having discounted genetic mutations or transgene-related interference as the underlying cause, we conclude that the failure of Daisy to develop a tail during embryonic/fetal development was most likely a consequence of epigenetic programming errors. That such an error manifests itself in a striking phenotype, such as tailessness, compellingly highlights the importance of epigenetic mechanisms in regulating gene activity to ensure correct implementation of developmental programs. Further, the recloning showed that this error was not stable and was either readily corrected during the reprogramming of the nucleus after SCNT or no longer present and already amended in Daisy's adult donor cells.

Taillessness is a rare congenital defect that has been observed in many different species (Anwar and Purohit, 2012, and references therein), including cattle produced by conventional reproduction. Although taillessness in sheep has been shown to have genetic origins (Basrur and Yadav, 1990), breeding trials with tailless cattle failed to establish a clear link between the absence of the tail and an underlying genetic cause (Gilmore and Fechheimer, 1957; Leipold et al., 1983).

A non-genetic cause could also provide an explanation for the occasional occurrence of taillessness in Chillingham Wild White cattle (Ingham and Widdows, 2005). Due to their long history of isolation and inbreeding, they show minimal genetic variation and detrimental alleles are expected to have been purged (Visscher et al., 2001; Williams et al., 2016). Thus, taillessness in these cattle is unlikely to be caused by a single recessive or closely linked group of genes (Ingham and Widdows, 2005). This strongly suggests that epigenetic errors could be responsible for a significant proportion of taillessness cases in the normal cattle population. In addition, Daisy's skeletal abnormalities closely resembled the deformities that have been described in several case studies as typical characteristics for tailless cattle in conventionally bred cattle of different breeds (Huston and Wearden, 1958).

Taillessness has previously been observed in cloned mice and cattle. In mice, SCNT cloning using cumulus donor cells resulted in the birth of two clones, one with a normal and the other one with a short, stubby tail (Balbach et al., 2007). Recloning of the stubby tail mouse yielded a day 16 fetus with a tail, but the tail tip had an atypical constriction. Remarkably, matings with wild-type mice for multiple generations were unable to completely restore a normal tail phenotype, which suggests a non-Mendelian mode of inheritance of the tail defect (Balbach et al., 2007).

In cattle, a tailless cloned transgenic calf was born after microinjection of a non-transgenic wild-type blastomere into an eight-cell embryo carrying a transgene (Chen et al., 2010). In the calf, the transgene cassette could no longer be detected, which suggests that the animal was entirely derived from the wild-type blastomere. This indicates that the lack of the tail was not caused by the transgene itself or its insertion into a particular locus that may be critical for tail development. The injected wild-type blastomere was derived from an embryo produced by *in vitro* fertilization. However, the authors did not provide information as to whether any of the two parental linages was associated with anury. Thus, it remains unclear as to whether the lack of a tail reported by Chen et al. was caused by genetic or epigenetic errors.

Taillessness in cattle produced by natural mating or conceived after *in vitro* fertilization is rare, and this certainly holds true for cloned cattle. The considerably lower number of reported cases of taillessness in cloned cattle and the unknown total number of cattle clones prevents us from a proper comparison between cloned and sexually conceived animals. Hence, we are unable to deduce whether cloning correlates with a greater incidence of tailless animals.

## References

[B1] Anwar S., and Purohit G.N. (2012). Rare congenital absence of tail (anury) and anus (atresia ani) in male camel (*Camelus dromedarius*) calf. Open Vet. J. 2, 69–71 26623295 PMC4655764

[B2] Balbach S.T., Jauch A., Bohm-Steuer B., Cavaleri F.M., Han Y.M., and Boiani M. (2007). Chromosome stability differs in cloned mouse embryos and derivative ES cells. Dev. Biol. 308, 309–321 17610862 10.1016/j.ydbio.2007.05.034

[B3] Basrur P.K., and Yadav B.R. (1990). Genetic diseases of sheep and goats. Vet. Clin. North Am. Food Anim. Pract. 6, 779–802 2245374 10.1016/s0749-0720(15)30846-x

[B4] Chen X., Wang J., Li R., Ding F., Li S., Zhang L., Dai Y., and Li N. (2010). Effect of microinjection of a single IVF-derived blastomere on the development of cloned embryos at the eight-cell stage in bovine. Cell. Reprogram. 12, 719–727 20969452 10.1089/cell.2010.0032

[B5] Chesley P., and Dunn L.C. (1936). The inheritance of taillessness (anury) in the house mouse. Genetics 21, 525–536 17246810 10.1093/genetics/21.5.525 PMC1208720

[B6] Gilmore L.O., and Fechheimer N. S. (1957). Tailless calves studied in attempt to determine cause of abnormality. Ohio Fm. Home Res. 42, 32–33

[B7] Greene H.J., Huston K., Leipold H.W. (1973). Taillessness in cattle. Giessener Beitr. Erbpath. Zuchthyg. 5, 158–170

[B8] Huston K., and Wearden S. (1958). Congenital taillessness in cattle. J. Dairy Sci. 41, 1359–1370

[B9] Ingham B., and Widdows A. (2005). Taillessness (anury) in a Chillingham White Wild calf. Trans. Nat. Hist. Soc. Northumbria 64, 169–172

[B10] Jabed A., Wagner S., McCracken J., Wells D.N., and Laible G. (2012). Targeted microRNA expression in dairy cattle directs production of beta-lactoglobulin-free, high-casein milk. Proc. Natl. Acad. Sci. U.S.A. 109, 16811–16816 23027958 10.1073/pnas.1210057109 PMC3479461

[B11] Kloppert B. (1986). Caudal, sacral and lumbar spinal column agenesis in cattle in Hessen and the associated congenital abnormality syndrome (caudo-rectouro-genital syndrome and anury-anophthalmia syndrome). Thesis, Justus-Liebig University, Giessen, Germany, p. 265

[B12] Leipold H.W., Huston K., and Dennis S.M. (1983). Bovine congenital defects. Adv. Vet. Sci. Comp. Med. 27, 197–271 6417989

[B13] Lotfi A., and Shahryar H.A. (2009). The case report of taillessness in Iranian female calf (a congenital abnormality). Asian J. Anim. Vet. Adv. 4, 47–51

[B14] Oback B., Wiersema A.T., Gaynor P., Laible G., Tucker F.C., Oliver J.E., Miller A.L., Troskie H.E., Wilson K.L., Forsyth J.T., et al. (2003). Cloned cattle derived from a novel zona-free embryo reconstruction system. Cloning Stem Cells 5, 3–12 12713696 10.1089/153623003321512111

[B15] Rieck G.W. (1966). [On taillessness in cattle]. Dtsch. Tierarztl. Wochenschr. 73, 80–85 6007716

[B16] Robinson R. (1993). Expressivity of the Manx gene in cats. J. Hered. 84, 170–172 8228170 10.1093/oxfordjournals.jhered.a111311

[B17] Visscher P.M., Smith D., Hall S.J.G., and Williams J.L. (2001). A viable herd of genetically uniform cattle. Nature 409, 303 11201728 10.1038/35053160

[B18] Williams J.L., Hall S.J.G., Del Corvo M., Ballingall K.T., Colli L., Ajmone Marsan P., and Biscarini F. (2016). Inbreeding and purging at the genomic level: the *Chillingham cattle* reveal extensive, non-random SNP heterozygosity. Anim. Genet. 47, 19–27 26559490 10.1111/age.12376

